# Notes and News

**Published:** 1904-06-11

**Authors:** 


### Notes and News.

Dr. Robert Ambrose, B.A., Member of Parliament for the Western Division of County Mayo has been elected President of the Incorporated Medical Practitioners' Association for the ensuing year.

His Majesty the King of Denmark, on the conclusion of the Conference at Copenhagen, received one representative from each country, who afterwards dined with the King and members of the Royal Family at the Palace. Dr. Nathan Raw, of Liverpool, had the honour of representing England.

Two new wings were opened at the Northampton General Hospital on Thursday by Earl Spencer. These much needed additions to the institution, which, with the general improvements connected with them, give Northampton practically a new hospital. This satisfactory fact resulted, it will be remembered, from the recommendations of Sir Henry Burdett, who was invited to advise the committee.

The managers of the Glasgow Royal Infirmary have remitted the complete plans for the reconstruction of this hospital to Sir Henry Burdett, and have associated with him Dr. Macintosh, of the Western Infirmary, Glasgow, "To examine and make suggestions as they may think advisable for their improvement in detail or as a working whole, bearing in mind that the general ground plan of the main ward blocks must not be departed from and that the number of beds provided (700) must not be diminished."

Isabella, Lady Lennard, Lady President of the Sevenoaks District of the League of Mercy, which is an auxiliary to King Edward's Hospital Fund for London, for the collection of small subscriptions from the metropolitan area and the surrounding counties, was present at an "At Home" last week at Brook House, Chislehurst, given by invitation of Mrs. Albin Fleming and Mrs. Nettleton Balme, Lady Vice-Presidents of the District. Among others who addressed the large number of ladies and gentlemen present was Dr. Norman Hay Forbes, a Vice-President of the Tonbridge District. The Sevenoaks District was instrumental in contributing last year £547 to the League of Mercy.

Inspector-General Herbert Mackay Ellis, R.N., has been selected for the appointment of Director-General of the Medical Department of the Royal Navy, in succession to Inspector-General Sir Henry Frederick Norbury, R.N., K.C.B., M.D., F.R.C.S., Honorary Surgeon to his Majesty, who will shortly relinquish the post.

At the invitation of Mrs. Hewlett, a lady vice-president of the Sevenoaks district, a drawing-room meeting was held on June 3 at Hillside House, Beckenham, to meet Isabella Lady Lennard, lady president of the Sevenoaks district, in order to further the objects of the League of Mercy in that neighbourhood. Mr. Hewlett presided, and an interesting address on the work of the League was given by Dr. H. J. Paterson, a vice-president of the East Marylebone district.

The Secretary for Scotland has received a memorial from the Royal College of Physicians of Edinburgh, which, whilst expressing approval of the provisions in the Scotch Education Bill, urges that the Education Department should have defined powers over the local bodies, and points out that medical inspectors should have the power of examining the health of children, and advising as to their physical and mental capacity for education, and also on all matters relating to school hygiene. The recommendations go as far as to advise that the school boards should have power to attend to the proper feeding of school children.

Interesting information has come home from the medical men forming the commission to the Congo for the purpose of studying trypanosomiasis. The expedition has been organised by the Liverpool School of Tropical Medicine, and the heavy expenses attending it have so far been defrayed by the King of the Belgians, Sir Alfred Jones, and other supporters of the Liverpool School. At Leopoldsville the Government built a hospital for the use of the expedition, where the members worked for four months under the most favourable conditions. Doctors Todd and J. E. Dutton have now proceeded up the Congo River to the less known regions.

The management of the Haddingtonshire Western District Infectious Diseases Hospital does not seem to be very satisfactory. We referred to the opening of patients' letters in a previous issue. This practice, it is announced, has now been put an end to. But matters are not as they should be in other directions. Surely it is remarkable that whilst the number of patients were practically the same, the expenditure on gas should increase by £50, as was stated at the recent meeting of the Town Council. The medical superintendent seems to have had no explanation to give. Public scrutiny is constantly directed to the management of our voluntary hospitals, and even in respect to Poor-law institutions public opinion has been beneficially exercised. But the fever hospitals are so circumstanced that information is not easily obtainable, and yet we believe that no part of our hospital system is so unsatisfactory, and in no direction is an absence of any standard of efficiency and economy so marked.

